# Suppurative Arthritis Following Acute Rheumatism

**Published:** 1877-09

**Authors:** V. P. Gibney


Suppurative Arthritis Following Acute Rheumatism.—*By V. P. Gibney, M.D.*—The specimen presented is the larger portion of the patella exfoliated from the right knee of a German lad nine years of age, who was admitted to the Hospital for the Ruptured and Crippled, March 8, 1877. The point of especial interest centering in the case is the fact that a rheumatic joint resulted in so profuse a suppuration. For acute rheumatism to often become chronic in the adult is not at all uncommon, for it to become chronic in the child is comparatively rare; but when it assumes a suppurative form, then an unusual interest attaches to the case and thorough analysis is demanded.

Whether the rheumatism in this instance was only one stage of the suppurative arthritis, or whether the rheumatism by its specific influence put the system in a favorable condition for the subsequent arthritis on the slightest provocation, is a question the solution of which I confess my inability to reach, and have accordingly brought it before the members for a full expression of opinion.

The history is as follows:

The boy belongs to a family of eight, only one of whom has died, the cause of this death reported as diphtheria. The family history, paternal and maternal, on a pretty thorough examination, is found unexceptionally good, no hereditary disease being discovered; the hygienic surroundings have been moderately fair; the health during infancy was not good, and a severe attack of pneumonia (from the history given) when he was one and a half years old, was followed within two or three months by a prolonged pertusis. From all these, however, he seemed to recover perfectly, and enjoyed good health uninterruptedly until the beginning of the present year. One day, about the middle of January, he came in from school complaining of a sense of weariness and pains throughout his bones; went to bed feverish, and on the following morning was unable to leave his bed. The joints became painful, began to swell, and within a few days a typical case of acute multiple rheumatic arthritis was present. A physician was in attendance for a week or ten days, convalescence set in, and about eight days after the doctor had discontinued his visits, seventeen days from the invasion, the right knee, which had not up to this time become entirely free from pain and a trifling amount of swelling, suddenly became very much enlarged, violent pain accompanied, and a severe relapse was thought to be imminent. A surgeon was called in, who prescribed rest and evaporating lotions, but spontaneous opening in a few days gave exit to a large quantity of offensive pus. Emaciation became marked, and on presentation at the hospital his condition was that of a patient well-nigh exhausted from some acute disease. He was totally unable to stand alone, the normal contour of the right knee was completely effected, the skin was bluish in appearance, the veins engorged and tortuous, while sloughing had taken place around two openings, leaving ugly ill-conditioned ulcers. Motion was preserved, *i. e.*, to as great an extent as the infiltrated periarbitic tissue would allow; no pain was elicited on a careful attempt to approximate the articular surfaces, the patella was movable, and in view of all the facts I made a diagnosis of periarthritis conseqent on acute rheumatism. The treatment was tonic medication, and simple dressing to the ulcers. I neglected to mention the peculiar expression given to the eyes. Such an expression is difficult to describe, but is just such as one observes in blondes who are decidedly strumous. The case progressed favorably until March 16th, when the swelling increased, the temperature rose to $103\frac{1}{2}^{\circ}$, and an additional abscess seemed to locate itself in the popliteal space. This was subsequently evacuated, and nothing of special note was observed till April 21st, when an extensive abscess filled the whole anterior portion of the thigh, a curious bone was seen protruding from the ulcer in prepatellary region; this was seized with a pair of dressing forceps, and the specimen now before you was easily removed. An apparatus was applied, the pus being first thoroughly evacuated and stimulants freely added to the tonics already being administered.

There is no question now about the joint being involved, and the course pursued by the disease seems clearly shown by the history as recorded, viz.: a suppurative periarthritis extending into the joint, necrosis, caries, etc.

The boy is at present in a better condition than the severity of the lesion would seem to warrant. The sac or sacs are being washed out twice daily with a solution of acid, carbolic. ʒi.–Oi., new abscesses are opened by incision as soon as they form, the limb is kept in a semi-extended position, and the prognosis on the whole is by no means as grave as it was a week ago.—*Medical Record.*

